# Transforming the German ICD-10 (ICD-10-GM) into Injury Severity Score (ISS)—Introducing a new method for automated re-coding

**DOI:** 10.1371/journal.pone.0257183

**Published:** 2021-09-10

**Authors:** Marcel Niemann, Sven Märdian, Pascal Niemann, Liv Tetteh, Serafeim Tsitsilonis, Karl F. Braun, Ulrich Stöckle, Frank Graef

**Affiliations:** 1 Center for Musculoskeletal Surgery, Charité – Universitätsmedizin Berlin, Corporate member of Freie Universität Berlin and Humboldt-Universität zu Berlin, Berlin, Germany; 2 Julius Wolff Institute for Biomechanics and Musculoskeletal Regeneration, Berlin Institute of Health at Charité – Universitätsmedizin Berlin, Berlin, Germany; 3 Department of Trauma Surgery, University of Munich, Munich, Germany; University Hospital Zurich, SWITZERLAND

## Abstract

**Background:**

While potentially timesaving, there is no program to automatically transform diagnosis codes of the ICD-10 German modification (ICD-10-GM) into the injury severity score (ISS).

**Objective:**

To develop a mapping method from ICD-10-GM into ICD-10 clinical modification (ICD-10-CM) to calculate the abbreviated injury scale (AIS) and ISS of each patient using the ICDPIC-R and to compare the manually and automatically calculated scores.

**Methods:**

Between January 2019 and June 2021, the most severe AIS of each body region and the ISS were manually calculated using medical documentation and radiology reports of all major trauma patients of a German level I trauma centre. The ICD-10-GM codes of these patients were exported from the electronic medical data system SAP, and a Java program was written to transform these into ICD-10-CM codes. Afterwards, the ICDPIC-R was used to automatically generate the most severe AIS of each body region and the ISS. The automatically and manually determined ISS and AIS scores were then tested for equivalence.

**Results:**

Statistical analysis revealed that the manually and automatically calculated ISS were significantly equivalent over the entire patient cohort. Further sub-group analysis, however, showed that equivalence could only be demonstrated for patients with an ISS between 16 and 24. Likewise, the highest AIS scores of each body region were not equal in the manually and automatically calculated group.

**Conclusion:**

Though achieving mapping results highly comparable to previous mapping methods of ICD-10-CM diagnosis codes, it is not unrestrictedly possible to automatically calculate the AIS and ISS using ICD-10-GM codes.

## Introduction

The Injury Severity Score (ISS) is used worldwide to assess trauma severity [1] and has been shown to correlate with mortality [2], hence, it is useful to grade injury severity to make outcomes comparable. The ISS primarily measures the anatomic injury and is again based on the Abbreviated Injury Scale (AIS). This, in turn, represents a standardized terminology to both describe and rank single injuries and their severity [3]. Finally, the ISS is calculated by the sum of squares of the AIS in each of the three most severely injured body regions [4]. According to the literature, severe polytrauma is defined as an ISS ≥ 16 [5], therefore, assigning the correct AIS score to a specific injury is of utmost importance to calculate a reliable ISS. However, this assignment remains a complicated process which requires adequate training. According to the Association for the Advancement of Automotive Medicine (AAAM), at least 14 hours of training are necessary [6].

Different methods of manual coding of diverging complexity have been published [6, 7]. However, even the so-called ’simplified’ coding methods remain challenging because they are time-consuming and do not allow for a straightforward assessment due to the complexity of the AIS coding process. This fact is even more pronounced when considering that large cohorts are needed to reach the calculated level of significance for most hypotheses in trauma research, according to the literature [8]. The AIS and ISS are not primarily assessed across trauma centres in the world. Nevertheless, coding according to the International Statistical Classification of Diseases and Related Health Problems (ICD) published by the World Health Organization (WHO) is commonly used for statistical reasons (e. g., cause of death) and currently available in its 10th revision (ICD-10). Since medical systems differ significantly throughout the world, various modifications of the classification exist (e. g., ICD-10-CM [Clinical Modification] used in the USA; ICD-10-GM [German modification] used in Germany and Switzerland). To date, different automated methods of re-coding the ICD-10-CM into AIS and ISS scores have been developed and published [9–17]. In times of ’Big Data’, these methods allow authors to easily transform huge datasets. It has been shown that these methods lead to reliable results compared to manual AIS and ISS coding [11–22]. In Germany, the ICD-10 classification is a significant parameter in the German Diagnosis Related Groups (G-DRG) system, which forms the medical system’s billing base. Thus, the ICD-10 had to be adapted to the German Health System (ICD-10-GM) [23], which is also used in Switzerland [24]. However, due to the classification changes, the above-mentioned automated methods cannot be utilised in these countries, leading to a complete loss of the potential benefits of such methods in trauma research.

Therefore, this study aimed to develop and validate a new automated mapping method to transfer ICD-10-GM into ICD-10-CM codes to use the ICDPIC-R [11] script to generate AIS and ISS scores from German ICD-10 diagnoses.

## Methods

The local ethics committee of the Charité–Universitätsmedizin Berlin approved the present study (application number EA2/028/21). Due to the retrospective nature of this study, additional patient consent was waived. The study was conducted in agreement with the principles of the seventh revision of the Declaration of Helsinki including its clarifications and with Good Clinical Practice Guidelines. Study data was retrieved from the electronic medical records archive (SAP ERP 6.0 EHP4, SAP AG, Walldorf, Germany). Prior to statistical analysis the data was fully anonymized.

We included all trauma patients that met the inclusion criteria (Table 1) who were admitted to the emergency room (ER) of a level I trauma centre from January 2020 through December 2020, in this retrospective study.

**Table 1:** Criteria for the Emergency Room (ER) protocol.

Injury mechanism:
• Pedestrian or cyclist injured with delta-v > 30 km/h
• Motorcyclist or car driver injured through high velocity
• Ejection from vehicle
• Death of any passenger
• Vehicle body damaged > 50 cm in depth
• Fall from ≥ 3 meters
• Blast injury
• Second-degree burn (°2b) of > 20% of the body surface area
• Crush injury
• Penetrating injury
Preclinical findings:
• Open traumatic brain injury
• Unstable thorax or open injury of the thorax
• Unstable pelvis
• ≥ One fracture of a long bone of the lower extremities
• Amputation proximal to the elbow or proximal to the knee
• Traumatic paraplegia
Physiology:
• Glasgow Coma Scale < 14
• Systolic blood pressure < 90 mmHg
• Respiratory rate < 10/min or > 29/min
• Peripheral oxygen saturation < 90%
• The need for mechanical ventilation
Other criteria not mentioned before:
• Strangulation
• An uncertain trauma mechanism or injury severity

Patients were included if at least one of the ER criteria was fulfilled.

AIS scores of all patients were evaluated manually using the simplified AIS version of 2005 published for the TraumaRegister DGU^®^ [7]. This is the standard AIS version utilized in Germany. Based on these documented scores, the ISS was calculated as described by medical professionals [1, 3, 7]. Furthermore, all ICD-10-GM codes of the study population were documented from the medical records. The following paragraph describes the differences in the ICD versions and the steps we passed through to re-code the ICD-10-GM data into the ICD-10-CM version.

**Fig 1:**
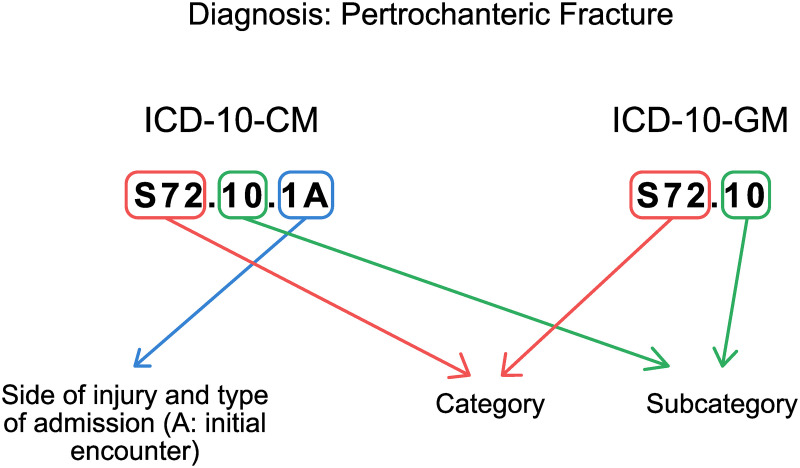
Visualisation of the differences between the ICD-10-CM and the ICD-10-GM coding system based on a pertrochanteric fracture example. ICD-10-CM = Clinical modification of the International Statistical Classification of Diseases and Related Health Problems, 10th revision. ICD-10-GM = German modification of the International Statistical Classification of Diseases and Related Health Problems, 10th revision.

Although both classifications are based on the ICD-10-WHO, they have a different level of detail (Fig 1), which is explained by the fact that the function of the ICD-10-CM version is statistical, as opposed to the German one, which is used for billing purposes. In summary, the ICD-10-CM results in an eight-digit code and the ICD-10-GM in a six-digit code. The two additional suffixes in the CM version do not influence the severity of the injury. The additional digit number seven in the CM version codes for varying information depending on the specific ICD-subcategory (e. g., injury side or patient’s gender) and can assume alphanumerical values (’0’, ’1’, ’2’, ’x’). Thus, when re-coding these ICD-codes, the specific subcategory must be taken into account to choose the correct values for digit number seven. Digit number eight codes for the type of admission (‘A’ = ‘initial encounter’, ‘D’ = ‘subsequent encounter’, ‘S’ = ‘sequela’). Because this digit does not influence injury severity, it was generally coded as ’A’. A further issue identified was that not all ICD-10-GM subcategories directly match their ICD-10-CM equivalent (e. g., ICD-10-GM: S22.03: fractures of the fifth and sixth thoracic vertebra, ICD-10-CM: S22.03: fracture of the third thoracic vertebra). Therefore, all ICD codes of the categories S00-S99 were manually reviewed for such differences between the two classification systems, and the need for re-coding was documented (e. g., all S22.03 of the ICD-10-GM were re-coded into S22.05 to match the ICD-10-CM version). An automated script then performed all changes. In a second step, the correct eight-digit format was applied to all codes by the script mentioned above, because the next step was to translate the resulting ICD-10-CM codes into the AIS and ISS scores. We used a script published elsewhere [11] to carry this into execution.

To automate the above-described tasks, a program was written in IntelliJ IDEA (IntelliJ IDEA Ultimate Edition for Windows, version 2020.1.4, JetBrains s. r. o., Prague, Czech Republic). The output file was imported into RStudio (RStudio Desktop Open Source Edition, version 1.2.5042, RStudio, MA, USA) and the General Equivalence Mapping (GEM) of the ICDPIC-R script [11] was used to calculate the corresponding ISS. The GEM method is based on the mapping tables provided in the ICDPIC Stata script [9], which re-codes ICD-10-CM into ICD-9-CM and matches the corresponding AIS. In some cases, one single ICD-10-CM diagnosis can be translated into multiple ICD-9-CM diagnoses. In these cases, the script offers to decide whether to translate the ICD-10-CM diagnosis into an ICD-9-CM diagnosis with a higher AIS (GEMmax) or a lower AIS (GEMmin). In the present study, GEMmax was used for further calculations. Finally, the ICDPIC-R calculated the ISS and the highest AIS for each body region. We compared the calculated results with the manually documented scores. For sub-analyses and the calculation of the inter-rater reliability, ISS scores were categorised as follows: Group 1 (ISS 0–8), group 2 (ISS 9–15), group 3 (ISS 16–24), group 4 (ISS 25–40), group 5 (ISS 41–49), and group 6 (ISS 50–75).

The patient selection process was performed as follows: In a first step, all patients who met the inclusion criteria (Table 1) and were admitted to the emergency room (ER) of a level I trauma centre from January 2020 through December 2020 were included in this retrospective study. Patients were then allotted into groups 1–6 and mean absolute differences as well as mean standard deviations of differences between the manual and automatic ISS methods were calculated for each of the groups. Sample size calculation was then conducted using the TOST power analysis for paired data with a power of 80%, an alpha error of 5%, and applying the highest standard deviation of differences found within one of the groups 1–6. The calculated sample size was performed to define the minimum number of patients required for the automatic script to work correctly.

Statistical analysis was performed using RStudio (RStudio, MA, USA). Data was analysed concerning normal distribution using histograms, QQ-plots, and mean/median. The data was tested for equivalence between groups using the two one-sided test (TOST) procedure. Equivalence was defined when the difference between two values lied within the equivalence bound of -3 to +3 and a corresponding null hypothesis significance test did not yield significance. Sample size calculations were conducted using the TOST power analysis for paired data. Inter-rater reliability was tested using weighted Kappa for multiple ordinal variables and two raters. A weighted Kappa value ≤ 0.2 was deemed ’poor’ agreement between both test methods, a value of 0.21–0.40 ’fair’ agreement, a value of 0.41–0.60 ’moderate’ agreement, a value of 0.61–0.80 ’good’ agreement, and a value of 0.81–1.0 ’excellent’ agreement. Differences between nonnormal data were tested for significance using the Wilcoxon-signed rank test. Bonferroni correction was applied for multiple testing. Unless stated otherwise, values are represented as mean ± SD. All *p*-values ≤ 0.05 were considered statistically significant.

## Results

In the first step, 520 patients from January to December 2020 met the inclusion criteria. Three hundred forty-five patients of this cohort were manually coded an ISS of 0–15, 80 patients were coded an ISS of 16–24 and 95 patients an ISS ≥ 25. Sample size calculation demonstrated that at least 138 patients were required for the script to work correctly in further subgroup analysis. In an effort to meet the sample size calculation for severely polytraumatized patients with an ISS ≥ 25, we additionally included all patients from January to December 2019 and from January to June 2021 with an ISS ≥ 16 in this study. This resulted in a total of 677 patients who met the inclusion criteria. Finally, 640 patients (m:w 465:175) were included in this study, 37 had to be excluded because ICD coding had not been completed upon the patients’ deaths (died within 24 hours after admission). In the final patient cohort, 341 patients had an ISS of 0–15, 155 patients an ISS of 16–24 and 144 patients an ISS of ≥ 25.

**Fig 2:**
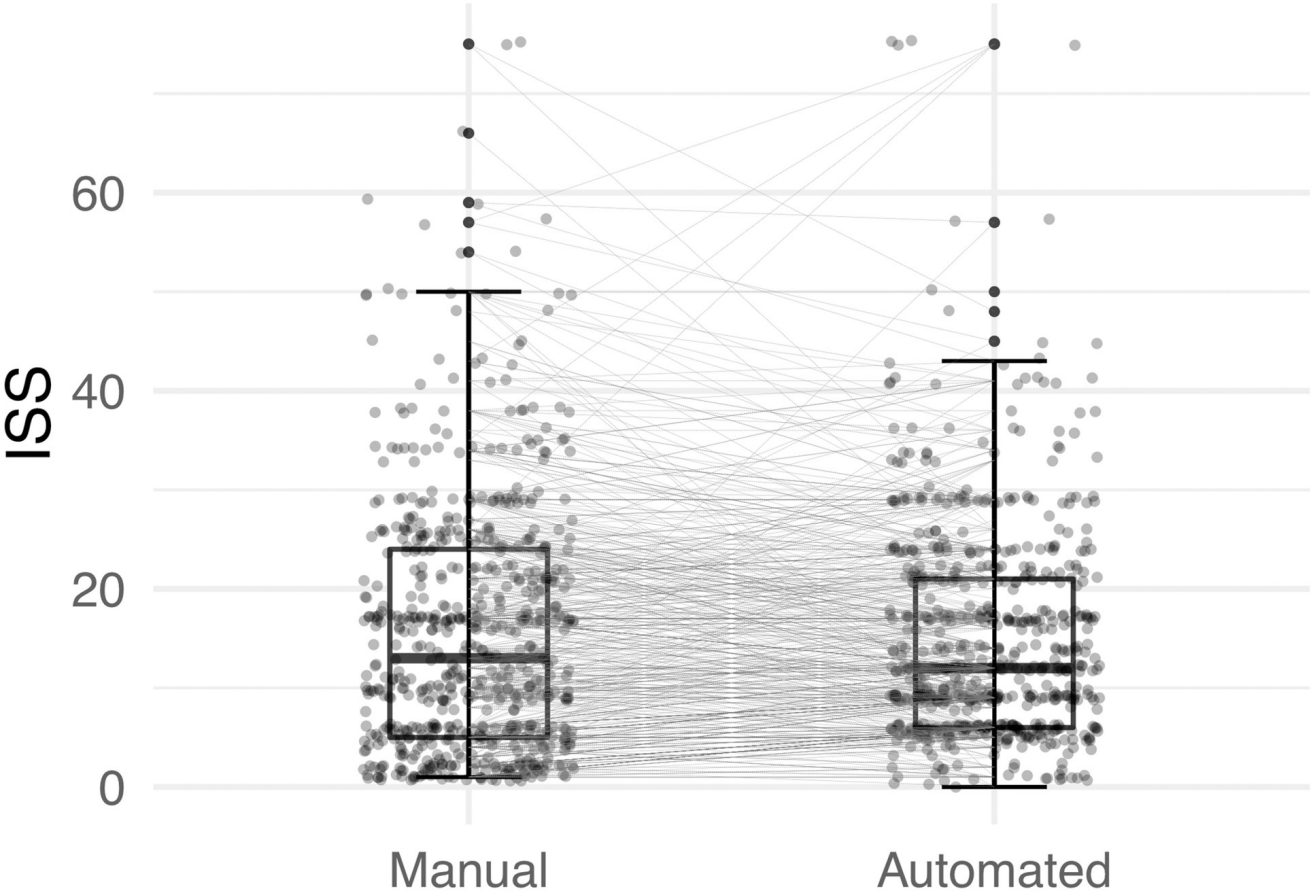
Boxplot with a median ISS of 13 (IQR 5, 24) by manual calculation and a median ISS of 12 (IQR 6, 21) by automated calculation. Each dot represents one measurement. Two measurements (one manually calculated, one automatically calculated) were available of each patient, which are graphically paired in this figure to visualise differences of the two ISS calculation methods. ISS = Injury Severity Score.

Mean age was 48.49 ± 22 years (range 2–96 years) at hospital admission. Manual calculation of the ISS revealed a median score of 13 (IQR 5, 24) and automatic calculation of the ISS using both scripts resulted in a median score of 12 (IQR 6, 21). Equivalence testing between the manually and automatically determined ISS demonstrated significance (*p* < 0.001, mean difference 0.44, 90% confidence interval (CI) -0.239–1.119). The results are visualised in Fig 2.

Subgroup analysis, however, revealed that the automatic ISS calculation from ICD-10 GM codes was only equivalent to the gold standard for patients with an ISS between 16 and 24 (Table 2).

**Table 2:** Equivalence testing between the automatic and manual ISS calculation.

ISS	n	TOST	NHST	Equivalence
0–8	219	p = 0.99	p < 0.001	No
9–15	122	p = 0.015	p = 0.005	No
16–24	155	p < 0.001	p = 0.058	Yes
25–40	114	p = 1.0	p < 0.001	No
41–49	13	p = 0.95	p < 0.05	No
50–75	17	p = 1.0	p < 0.001	No
0–75	640	p < 0.001	p = 0.286	Yes

Equivalence was present if the TOST was significant and the NHST was not significant. TOST = two one-sided test. NHST = null hypothesis significance test.

**Fig 3:**
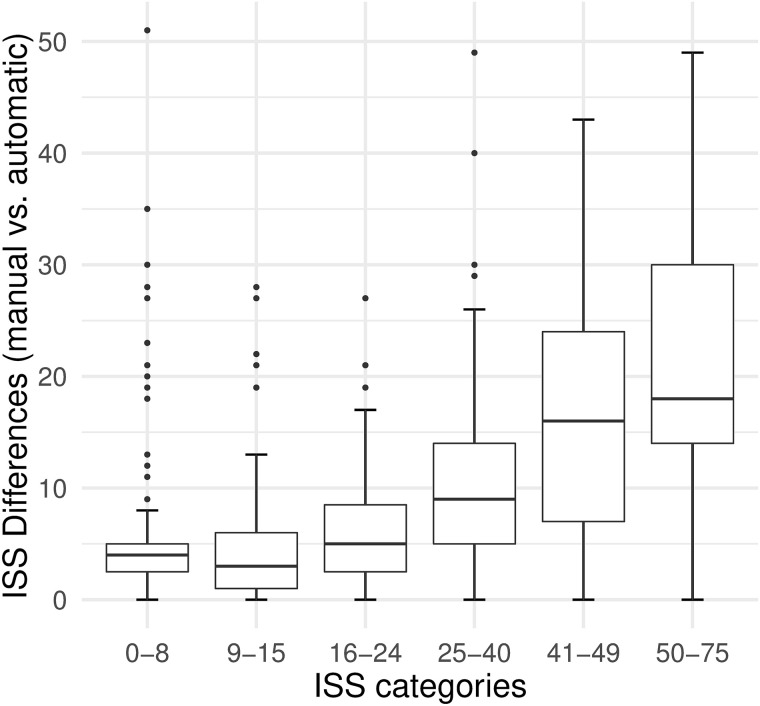
Individual absolute differences between the manual and the automatic ISS calculation. Group 1: ISS 0–8. Group 2: ISS 9–15. Group 3: ISS 16–24. Group 4: ISS 25–40. Group 5: ISS 41–49. Group 6: ISS 50–75. ISS = Injury Severity Score.

Analysis of the total absolute differences between automatic and manual calculation revealed that higher ISS scores were associated with higher differences between both methods (Fig 3).

Calculation of the ISS’s overall inter-rater reliability revealed a moderate concordance of the results (weighted Kappa = 0.47; Table 3). The inter-rater reliability of the AIS was ’excellent’ regarding the extremities, ’good’ concerning face and thorax, ’moderate’ regarding head and neck and the abdomen, and ’poor’ when considering external injuries (Table 4).

**Table 3:** Cross-tabulation of the automatically and manually calculated ISS.

Manually calculated ISS	Automatically calculated ISS					
	0–8	9–15	16–25	25–40	41–49	50–75
0–8	126	73	11	7	1	1
9–15	25	66	25	6	0	0
16–25	13	43	75	22	2	0
25–40	8	22	48	28	6	2
41–49	1	1	2	5	3	1
50–75	2	0	2	6	4	3

Overall, weighted Kappa was 0.47, which was deemed a ’moderate’ agreement between both methods. ISS = Injury severity score.

**Table 4:** Results of the inter-rater reliability and equivalence testing between manually and automatically AIS scores of each body region.

AIS category	Manual method: Mean highest AIS ± SD	Automatic method: Mean highest AIS ± SD	Weighted Kappa	TOST	NHST	Equivalence
Head and neck	1.94 ± 1.58 range (0–6)	2.19 ± 1.27 range (0–6)	0.449	p < 0.001	p < 0.001	No
Face	0.40 ± 0.79 range (0–3)	0.31 ± 0.62 (range 0–2)	0.578	p < 0.001	p < 0.001	No
Thorax	1.05 ± 1.52 (range 0–5)	1.13 ± 1.29 (range 0–5)	0.623	p < 0.001	p < 0.05	No
Abdomen	0.54 ± 1.14 (range 0–5)	0.90 ± 1.01 (range 0–5)	0.435	p < 0.001	p < 0.001	No
Extremities	1.18 ± 1.39 (range 0–5)	1.09 ± 1.29 (range 0–6)	0.757	p < 0.001	p < 0.05	No
External	0.75 ± 0.73 (range 0–5)	1.09 ± 0.86 (range 0–5)	0.0927	p < 0.001	p < 0.001	No

Inter-rater reliability was calculated using weighted Kappa for multiple ordinal variables and two raters. A weighted Kappa value ≤ 0.2 was deemed ’poor’ agreement between both test methods, a value of 0.21–0.40 ’fair’ agreement, a value of 0.41–0.60 ’moderate’ agreement, a value of 0.61–0.80 ’good’ agreement, and a value of 0.81–1.0 ’excellent’ agreement. Equivalence testing was performed with the two one-sided test (TOST), a lower bound of -3, an upper bound of +3 and an alpha error of 0.05. Equivalence was present if the TOST was significant and the null hypothesis significance testing (NHST) not significant. AIS = Abbreviated Injury Scale.

## Discussion

The present study describes a new and innovative method for the automated transformation of ICD-10-GM codes into ISS and AIS scores. We demonstrated that this method reveals ISS and AIS calculations with moderate agreement when compared to manual coding but fails to achieve reliable individual mapping results.

Methods for an automated calculation of the AIS and ISS based on the ICD-10-CM have been published previously [9–17] and have been used ever since [11–22]. Nevertheless, due to the underlying database of the ICD-10-CM, these methods can only be used in countries where this coding system is deployed, mainly the United States of America [25]. Hence, the present study focused on developing a method to transfer the German version of the ICD-10 into the ICD-10-CM version. The most significant issue when transferring the ICD-10-GM into ICD-10-CM is the difference in the code format. As mentioned before, ICD-10-GM is a six-digit code, whereas the ICD-10-CM consists of eight digits. Exemplary, the cyphers ’S06.0’ through ’S06.9’ are used to code intracranial injuries. In fact, the ICD-10-GM subclassifies 27 diagnoses, and the ICD-10-CM subclassifies 688 diagnoses when considering all ’S06.- ’ codes. A patient suffering from a subdural haematoma is encrypted with the ’S06.5’ code in the ICD-10-GM system. The ICD-10-CM system offers nine different codes for the same injury (’S06.5X0A’ to ’S06.5X9A’). Each subcategory adds information about the duration of consciousness (e. g. ’S06.5X5A’ = traumatic subdural haemorrhage with loss of consciousness greater than 24 hours with return to the pre-existing level of conscious, initial encounter). With these subcategories, the ICD-10-CM coding system gets closer to the AIS when compared to the ICD-10-GM coding system. The ICD-10-GM version adds some information by a second code (’S06.7-!’). Our method takes these differences into account and matches the ICD-10-GM codes and the secondary detail codes as much as possible to their corresponding ICD-10-CM code.

**Fig 4:**
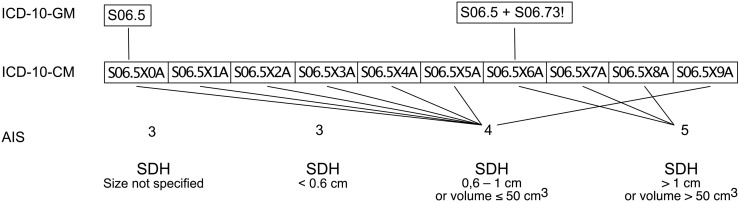
Exemplary visualisation of the mapping process from ICD-10-GM into AIS codes. The necessary information to sufficiently encode AIS scores is not displayed in the ICD system in general. ICD-10-GM = German modification of the International Statistical Classification of Diseases and Related Health Problems 10th revision. ICD-10-CM = Clinical modification of the International Statistical Classification of Diseases and Related Health Problems 10th revision. SDH = Subdural haematoma. AIS = Abbreviated injury scale.

Even though the script presented here allows for a translation of ICD-10-GM codes into ICD-10-CM codes, the further transfer of ICD-10-CM into AIS codes remains inaccurate (Fig 4). The differences of the coded information between the ICD-10 and the AIS itself are major limiting factors. As shown in Fig 4, the AIS of a subdural haematoma (SDH) ranges from three to five depending on the haematoma size. However, this information is not included in the ICD-10 system. The ICDPIC-R script translates all SDH diagnoses into an AIS of 4, except for cases when unconsciousness occurs for more than 24 hours without returning to the previous level of consciousness. In this case, the AIS scores 5.

Studies published earlier could demonstrate that automated transfer methods from ICD-10-CM codes to ISS were overall statistically equivalent to the gold standard of manually determining the ISS [11–22]. These results were replicable in our study. Furthermore, our absolute mean differences between the automatically and manually calculated ISS (6.96 ± 7.73) were comparable to those stated in the literature, e. g. of Di Bartolomeo et al. (6.36 ± 14.45) [19]. However, none of these previous studies performed further analysis for ISS subgroups or individualized ISS values. An overall agreement may be explained by a smaller impact of extreme values and data concentration closely near the mean in suffitiently large cohorts. While equivalence analysis revealed moderate accordance (weighted Kappa = 0.47) between both methods in our study, some data published showed ranges of weighted Kappa between 0.44 to 0.78 [11–14, 18, 20–21]. Despite an overall equivalence between methods and a moderate overall agreement between methods (weighted kappa of 0.47), subgroup analyses did not reach satisfying results. Equivalence was only observed in patients with an ISS between 16 and 24. However, we were the first authors to add ISS subgroup analysis to reach a higher level of evidence concerning our data interpretation. When only comparing the previously published study data, the results of our study are highly comparable to those [11–14, 18, 20–21]. Therefore, we achieved the goal to close the gap between ICD-10-GM and ICD-10-CM diagnosis codes. Nonetheless, neither our nor any other previously published automated mapping method is reliable when compared to manual coding. Accordingly, manual ISS coding remains the gold standard. In times of Big Data and progressive digitalization in health care, however, such digitalization attempts are highly needed. For such attempts, the standard coding systems used at the hospitals need to be specified in order to meet injury pattern and injury severity. Up until now, trauma diagnoses are not sufficiently represented in any ICD system and the ICD coding systems are too inaccurate when compared to the highly precise injury classification of the AIS.

Lastly, this is the first time an automated method for transferring ICD-10-GM data into ISS and AIS scores is validated. Hartensuer et al. investigated the technical feasibility of a mapping method to re-code ICD-10-GM into ISS but did not provide any statistical data to sufficiently compare their results with the outcomes presented here [16]. Future authors may use our attempt to automatize future ICD GM versions directly into ISS and AIS scores.

This study has some limitations. The ICD-10-GM is a simplified version of the ICD-10-CM implicating that the level of information coded in the ICD-10-CM is not available. This is because the ICD-10-GM is used as an accounting tool, whereas the ICD-10-CM serves as the basis for statistical analysis in the health system. Besides, the manual mapping was performed according to a simplified algorithm published elsewhere [7]. Although being widely used, the mapping is less specific compared to the original AIS mapping [6]. Furthermore, our mapping method is limited to the ICD-10-GM version and, thus, not usable in countries were another ICD-version is used. Nevertheless, we intended to develop a method for this ICD-10 version exclusively and enable other research groups to use it combined with the previously published algorithms for the final re-coding into AIS and ISS [9–17]. Future authors may consider adapting their scripts to output injury severity in the AIS categories besides the ISS body regions, which are needed for the ISS calculation. The output of injury severity in different AIS categories may add important additional information about the study cohort. Unfortunately, we were not able to do so. We aimed to close the gap between ICD-10-GM and ICD-10-CM diagnosis codes and depended on the ICDPIC-R to transform the AIS values into the ISS. The ICDPIC-R cannot output injury severity in accordance with the AIS chapters. Also, the AIS version of 2005 was used in this study. The reason for this was, that the polytrauma data base from the German trauma association (TraumaRegister DGU^®^) uses a simplified method to determine AIS and ISS codes based on the AIS from 2005. Because this study aimed to automatically derive AIS and ISS codes from the German ICD-10 coding system and in order to guarantee comparability, we decided to use the AIS 2005 version. The most recent AIS version would have been the 2015 version. Lastly, the used definition of polytrauma may be critizised. In line with previous authors, we have used the ISS-based polytrauma definition. Utilizing this definition, polytrauma patients may be identified when the total injury severity exceeds an ISS of 15. Recently, authors expressed concern about the inter-rater reliability of such a definition and demonstrated that inter-rater reliability of the Berlin Definition goes beyond that ISS based definition [26]. However, the decision to use the ISS polytrauma definition for our study was made to ensure comparability to previous studies.

## Conclusion

For the first time, this study describes a method to transfer ICD-10-GM codes into AIS and ISS scores. Even though providing results which were highly comparable to previous mapping tools, the presented mapping tool failed at achieving accurate results for individual ISS values when compared to manual coding. Therefore, manual ISS coding of an AAST certified investigator remains the gold standard. Unless the utilized coding systems of hospitals are not enormously specified to suffitietly meet coding standards in trauma, automated ICD mapping tools cannot be expected to be an adequate substitute for manual coding.

## Supporting information

S1 Program (JAVA)

S1 File (PDF)

S1 Dataset (XLSX)
