# Supplementary material for: Transforming the German ICD-10 (ICD-10-GM) into Injury Severity Score (ISS)—Introducing a new method for automated re-coding
Source: PLoS One. 2021 Sep 10;16(9):e0257183. doi: 10.1371/journal.pone.0257183 (PMC8432850; doi:10.1371/journal.pone.0257183)
Supplement: S1 File — (PDF) [file pone.0257183.s002.pdf]

Introduction

This is a user guide for the recently published mapping program from the German modification of the International Classification of Diseases 10th revision (ICD-10-GM) codes into the clinical modification of the ICD-10 (ICD-10-CM). Herewith, already published programs transforming ICD-10-CM into Injury Severity Scores (ISS) may be used. This Java™ program may be used both on macOS and Microsoft Windows. Necessarily, users need to install Java™ (Java™, Oracle Corporation, Austin, TX, USA) in order to run the program.

Guide

This guide and all illustrations included in it are based on Terminal (Version 2.11), macOS (Version 11.1), and Java™ (Java™ Standard Edition, version 15.0.1, Oracle Corporation, Austin, TX, USA). However, users may run the program in Terminal on macOS as well as in the command processor shell or in PowerShell on Windows without any modifications to the procedure shown below.

After starting Terminal, users can start the program using the command 'java' followed by the program path. This procedure requires at least Java™ 11. In cases of any earlier version of Java™ installed, users need to use the command 'javac' followed by the program path in order to compile the program. Afterwards, the command 'java' followed by the program name can be used to start the program.

Firstly, users are asked to define the first column of the subsequently imported comma-separated values (csv) data sheet including an ICD-10-GM code. In this illustration, the first ICD-10-GM was in column 'm'. Therefore, the input was '13'.

Secondly, users are asked to decide on the extent of information they need about the transformation process. The option 'short information' compiles a txt file in which diagnosis

codes are included that could not be directly translated from ICD-10-GM into ICD-10-CM. The output file includes information about the process the program used to find a matching ICD-10-CM diagnosis as well as the baseline ICD-10-GM and the resulting ICD-10-CM code. The option ‘full information’ compiles a txt file in which all baseline ICD-10-GM and the individually resulting ICD-10-CM codes are included as well as information about the process the program used to find a matching ICD-10-GM code. The option ‘no information’ does not compile any txt output file. Figure 1.

a

b

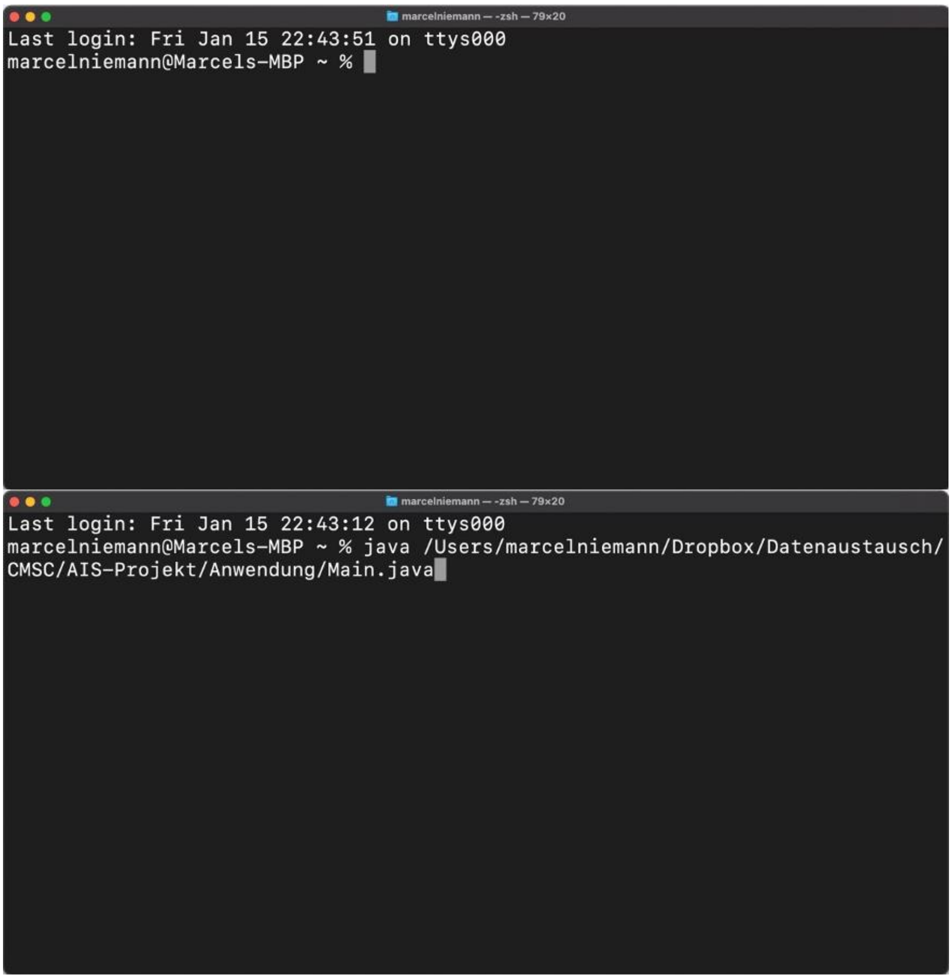

c

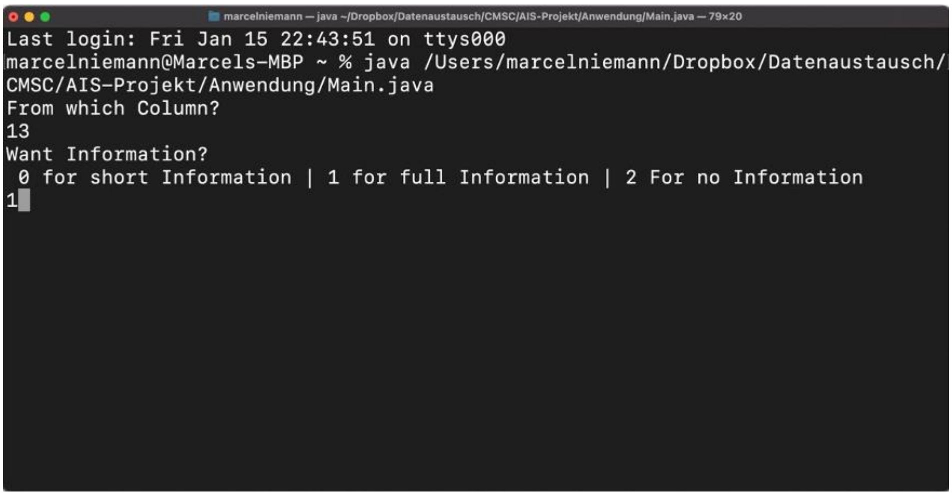

Figure 1 Program sequence in Terminal (Version 2.11) on macOS (Version 11.1). **a** depicts the Terminal after its start. **b** depicts the command process needed to start the transformation program. **c** depicts the required input after starting the transformation program.

Afterwards, a window automatically opens in which the user needs to choose a reference list, a baseline csv file, an output txt file, and an output csv file.

The reference list may be any csv file including ICD-10-CM diagnosis as a transformation target. The diagnosis list used in the original publication of this transformation program are included in the appendix of the publication.

The baseline file is any csv file including ICD-10-GM codes that users want to transform into ICD-10-CM codes.

The output txt file can be any existing txt file. After running the program, this file includes the aforementioned information about the transformation process. The user does not need to save any empty txt file before using this program. In case the user selects any txt file not existing (c. f. figure 2c), the program creates this file.

The output csv file is the output file resulting of the baseline file after transforming the included ICD-10-GM into ICD-10-CM codes. As described, file not existing when selected by the user are created by the program (c. f. figure 2d).

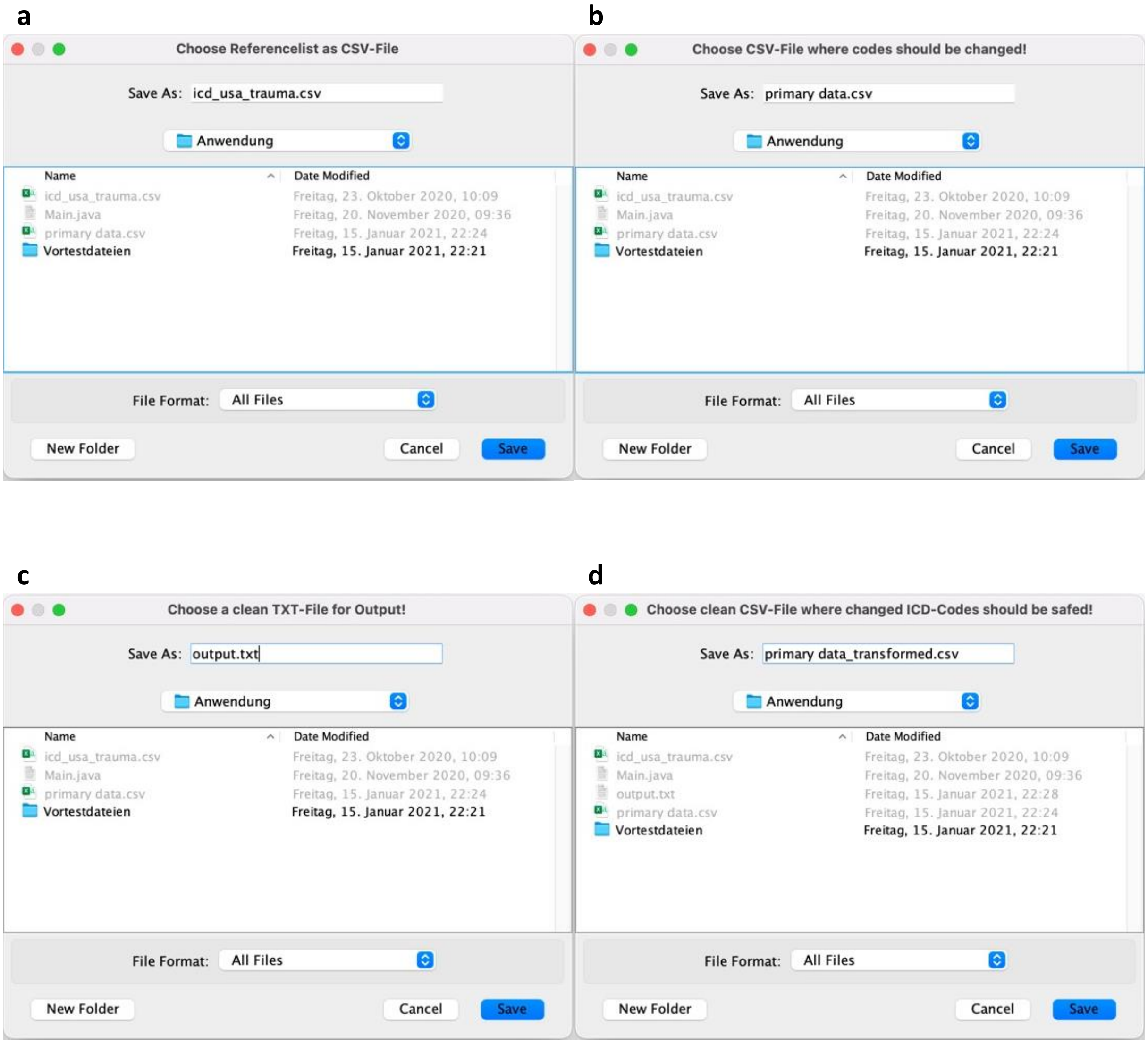

Figure 2 Program sequence following the Terminal sequence. Successively, users need to select a reference list (**a**), a baseline csv file (**b**), an output txt file (**c**), and an output csv file (**d**).

Results

After selecting every file, all windows automatically close and files not existing are created. A possibly resulting output txt file when choosing 'full information' is shown in figure 3. Figure 4 depicts a comparison between the baseline and the output csv file after running the program as explained.

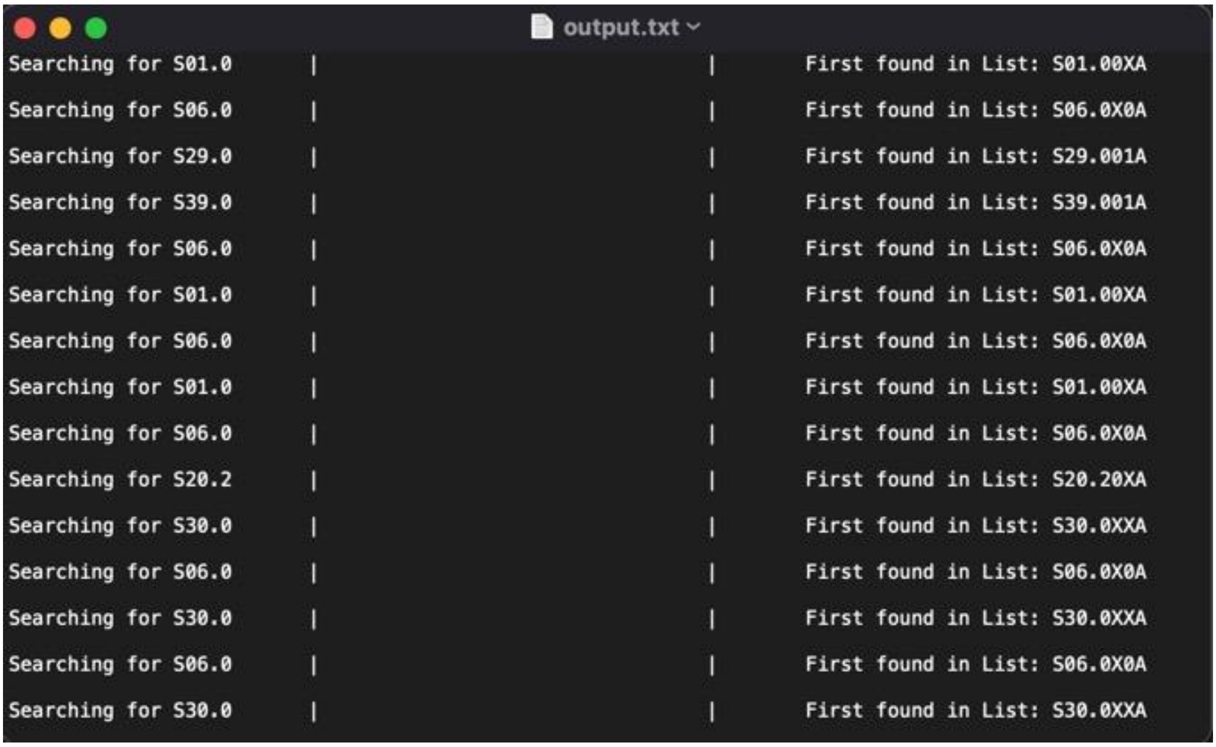
output.txt

```
Searching for S01.0    |    |    First found in List: S01.00XA
Searching for S06.0    |    |    First found in List: S06.0X0A
Searching for S29.0    |    |    First found in List: S29.001A
Searching for S39.0    |    |    First found in List: S39.001A
Searching for S06.0    |    |    First found in List: S06.0X0A
Searching for S01.0    |    |    First found in List: S01.00XA
Searching for S06.0    |    |    First found in List: S06.0X0A
Searching for S01.0    |    |    First found in List: S01.00XA
Searching for S06.0    |    |    First found in List: S06.0X0A
Searching for S20.2    |    |    First found in List: S20.20XA
Searching for S30.0    |    |    First found in List: S30.0XXA
Searching for S06.0    |    |    First found in List: S06.0X0A
Searching for S30.0    |    |    First found in List: S30.0XXA
Searching for S06.0    |    |    First found in List: S06.0X0A
Searching for S30.0    |    |    First found in List: S30.0XXA
```

Figure 3 Output txt file for illustrations.

a

M	N	O	P	Q	R	S	T	U	V	W	X	Y	Z	AA	AB	AC
S01.0	S06.0	S29.9	S39.9		S06.0		F10.0	F10.2	J96.01	S01.0	S06.0			S01.0		
S06.0	S30.0			S06.0	S30.0		O09.4	S30.0	W64.9	Z34						Z35.1
S06.0	S32.89	S82.41			S06.0	S11.84	S12.1	S13.4	S20.2	S30.1	S31.84	S31.84	S32.4	S32.5	S80.0	S80.1
S06.0	S22.20	S29.9	S39.9	S52.51		E03.8	S06.0	S20.2	S30.1	S51.85	S52.50	S80.81	S80.81	Z04.1	Z23.5	
S06.0		J69.0	S06.0		A41.8	E51.2	E51.9	E86	E87.6	F05.0	F10.0	F10.2	F10.4	F10.6	I10.90	J15.5
S06.0	S42.3		D90	S00.05	S01.80	S06.0	S06.79	S20.2	S30.1	S41.84	S42.3	Z88.0	Z92.1		C61	E03.8
R42	S00.31	S06.0	S63.60	S80.81		I48.0	R55	S00.31	S00.81	S00.85	S06.0	S63.60	S80.81	Z92.1		S06.0
G40.1	G40.9		G40.1		G40.9		S01.0				G40.1	R41.2	S00.1	S01.0	S06.6	S06.70
S06.5		S06.5		S06.5			G93.6	J69.0	S01.84	S02.1	S06.5	S31.84	S32.03	U69.01		
S01.21	S06.0	S29.9	S39.9	S80.1		R40.0	S06.0	S06.79	Z23.5			S06.0		S02.2		
S01.83	S06.0	S06.79	S06.9		F10.0	J96.00	S06.5	S06.79				D69.40	S01.0	S06.0	S06.5	S06.79
S06.0	S21.83	S27.0	S61.9	T14.1		S06.0	S11.02	S11.9	S21.83	S27.0	S29.9	S61.9	T14.1	W49.9	Z91.8	
I46.0	U69.13		S00.05	S06.0	S06.79		E87.6	I25.13	I25.5	I27.28	I44.7	I48.0	I49.8	I65.2	J96.00	R57.0
N17.99	S72.10		L89.08	S71.85	S72.10		B96.8	E83.38	F05.0	I10.90	I48.0	I71.2	J45.1	J96.00	J96.01	K56.7
S06.0	S06.79	S20.2	S30.1	S81.84	S82.5	Z04.1	Z46.7		S81.84	S82.5	V99					S81.84
I61.9	S06.0		S06.0	S06.33	S06.70		D69.58	F00.2	G30.8	I10.90	S06.0	S06.1	S06.33	S06.70	S06.70	S06.70
E86	G40.8		E86	G40.8	G40.8	I10.00	I48.0					B96.2	E87.6	G40.8	J15.2	J15.6
S06.9	S29.9	S39.9	S56.3		S20.2	S21.84	S22.42	S30.1		S21.84	S22.42				E27.4	I10.90
V99	Z03.3		S06.0	S06.79	S20.2	V99	Z88.8		Z03.3							
S20.2	T09.3		S20.2	T09.3			G55.3	M48.06						M62.47	M62.57	
S06.0	S06.79		S06.0	V99			E11.90	I10.90	I25.10	S06.0	S20.2	S30.1	S80.81	Z04.1	Z92.2	
T11.1		S56.1		J96.00	S54.1	S55.1	S56.1			S55.1	S66.1					S54.1
J94.2		S21.83	S27.2	Y09.9		S27.2			S21.83	S27.2						
S52.9		B96.6	G40.8	H91.3	S00.81	S01.84	S02.2	S20.2	S30.1	S52.50	T89.02	Z11	Z23.5		E03.8	M79.83
S32.89		S06.0	S31.84	S31.84	S32.1	S32.5		S31.84	S32.5		S06.0			S32.7		
S06.0	S30.0	S39.9		S00.85	S06.0	S20.2	S30.1		S06.0			S00.05	S06.0	S06.79	V99	Z04.1
S02.5	S06.0	S29.9	S39.9	S82.18			S01.88	S02.5	S06.0	S20.2	S30.1				F10.0	R41.2
S01.0	S06.0	S12.24	S12.25		S01.0	S06.0		S11.84	S11.84	S12.24	S12.25			S01.0		
G41.0	S06.0			G40.8			G41.0									
S29.9	S36.12	S39.9		L89.11	S31.83	S36.11		B37.1	B95.2	B96.2	B96.2	B96.5	B96.7	D62	D65.0	D68.4
S20.2	S30.1	S81.85	S81.88	S82.38	S82.38			M79.86	S06.0	S81.88	S82.38	Z46.7	Z47.8	Z74.0	Z74.1	

primary data

b

M	N	O	P	Q	R	S	T	U	V	W	X	Y	Z	AA	AB	AC
S01.00XA	S06.0X0A	S29.001A	S39.001A		S06.0X0A		F10.0	F10.2	J96.01	S01.00XA	S06.0X0A			S01.00XA		
S06.0X0A	S30.0XXA			S06.0X0A	S30.0XXA		O09.4	S30.0XXA	W64.9	Z34						Z35.1
S06.0X0A	S32.89XA	S82.401A			S06.0X0A	S11.84	S12.100A	S13.4XXA	S20.20XA	S30.0XXA	S31.84	S31.84	S32.401A	S32.501A	S80.00XA	S80.10XA
S06.0X0A	S22.20XA	S29.001A	S39.001A	S52.531A		E03.8	S06.0X0A	S20.20XA	S30.0XXA	S51.85	S52.501A	S80.811A	S80.811A	Z04.1	Z23.5	
S06.0X0A		J69.0	S06.0X0A		A41.8	E51.2	E51.9	E86	E87.6	F05.0	F10.0	F10.2	F10.4	F10.6	I10.90	J15.5
S06.0X0A	S42.301A		D90	S00.05XA	S01.80XA	S06.0X0A	S06.0X0A	S20.20XA	S30.0XXA	S41.84	S42.301A	Z88.0	Z92.1		C61	E03.8
R42	S00.31XA	S06.0X0A	S63.601A	S80.811A		I48.0	R55	S00.31XA	S00.81XA	S00.85XA	S06.0X0A	S63.601A	S80.811A	Z92.1		S06.0X0A
G40.1	G40.9		G40.1		G40.9		S01.00XA				G40.1	R41.2	S00.10XA	S01.00XA	S06.6X0A	S06.0X0A
S06.5X0A		S06.5X0A		S06.5X0A			G93.6	J69.0	S01.84XA	S02.101A	S06.5X0A	S31.84	S32.030A	U69.01		
S01.21XA	S06.0X0A	S29.001A	S39.001A	S80.10XA		R40.0	S06.0X0A	S06.0X0A	Z23.5			S06.0X0A		S02.2XXA		
S01.83XA	S06.0X0A	S06.0X0A	S06.9X0A		F10.0	J96.00	S06.5X0A	S06.0X0A				D69.40	S01.00XA	S06.0X0A	S06.5X0A	S06.0X0A
S06.0X0A	S21.83	S27.0XXA	S61.001A	T14.1		S06.0X0A	S11.021A	S11.90XA	S21.83	S27.0XXA	S29.001A	S61.001A	T14.1	W49.9	Z91.8	
I46.0	U69.13		S00.05XA	S06.0X0A	S06.0X0A		E87.6	I25.13	I25.5	I27.28	I44.7	I48.0	I49.8	I65.2	J96.00	R57.0
N17.99	S72.101A		L89.08	S71.85	S72.101A		B96.8	E83.38	F05.0	I10.90	I48.0	I71.2	J45.1	J96.00	J96.01	K56.7
S06.0X0A	S06.0X0A	S20.20XA	S30.0XXA	S81.84	S82.51XA	Z04.1	Z46.7		S81.84	S82.51XA	V99					S81.84
I61.9	S06.0X0A		S06.0X0A	S06.330A	S06.0X0A		D69.58	F00.2	G30.8	I10.90	S06.0X0A	S06.1X0A	S06.330A	S06.0X0A	S06.0X0A	S06.0X0A
E86	G40.8		E86	G40.8	G40.8	I10.00	I48.0					B96.2	E87.6	G40.8	J15.2	J15.6
S06.9X0A	S29.001A	S39.001A	S56.301A		S20.20XA	S21.84	S22.49XA	S30.0XXA		S21.84	S22.49XA				E27.4	I10.90
V99	Z03.3		S06.0X0A	S06.0X0A	S20.20XA	V99	Z88.8		Z03.3							
S20.20XA	T09.3		S20.20XA	T09.3			G55.3	M48.06						M62.47	M62.57	
S06.0X0A	S06.0X0A		S06.0X0A	V99			E11.90	I10.90	I25.10	S06.0X0A	S20.20XA	S30.0XXA	S80.811A	Z04.1	Z92.2	
T11.1		S56.101A		J96.00	S54.10XA	S55.101A	S56.101A			S55.101A	S66.100A					S54.10XA
J94.2		S21.83	S27.2XXA	Y09.9		S27.2XXA			S21.83	S27.2XXA						
S52.501A		B96.6	G40.8	H91.3	S00.81XA	S01.84XA	S02.2XXA	S20.20XA	S30.0XXA	S52.501A	T89.02	Z11	Z23.5		E03.8	M79.83
S32.89XA		S06.0X0A	S31.84	S31.84	S32.10XA	S32.501A		S31.84	S32.501A		S06.0X0A			S32.810A		
S06.0X0A	S30.0XXA	S39.001A		S00.85XA	S06.0X0A	S20.20XA	S30.0XXA		S06.0X0A			S00.05XA	S06.0X0A	S06.0X0A	V99	Z04.1
S02.5XXA	S06.0X0A	S29.001A	S39.001A	S82.101A			S01.80XA	S02.5XXA	S06.0X0A	S20.20XA	S30.0XXA				F10.0	R41.2
S01.00XA	S06.0X0A	S12.500A	S12.600A		S01.00XA	S06.0X0A		S11.84	S11.84	S12.500A	S12.600A			S01.00XA		
G41.0	S06.0X0A			G40.8			G41.0									
S29.001A	S36.113A	S39.001A		L89.11	S31.83	S36.112A		B37.1	B95.2	B96.2	B96.2	B96.5	B96.7	D62	D65.0	D68.4
S20.20XA	S30.0XXA	S81.85	S81.88	S82.301A	S82.301A			M79.86	S06.0X0A	S81.88	S82.301A	Z46.7	Z47.8	Z74.0	Z74.1	

primary data_transformed

Figure 4 Comparison between the baseline and the output data. **a** depicts the baseline csv data. **b** depicts the output csv data.

Annotations

The program published is an open-source software. Any remarks or troubleshooting requests may be sent to marcel.niemann@charite.de.
